# An Assessment of the Validity of an Audio-Video Method of Food Journaling for Dietary Quantity and Quality

**DOI:** 10.1155/2019/9839320

**Published:** 2019-03-26

**Authors:** Emily Jago, Alain P. Gauthier, Ann Pegoraro, Sandra C. Dorman

**Affiliations:** ^1^Laurentian University, Sudbury, Canada; ^2^Director Centre for Rural and Northern Health Research, Laurentian University, Sudbury, Canada; ^3^School of Human Kinetics, Laurentian University, Sudbury, Canada; ^4^Director Centre for Research Occupational Safety and Health, Laurentian University, Sudbury, Canada

## Abstract

**Objective:**

To validate an audio-video (AV) method of food journaling, in a free-living scenario, compared to direct, weighed food assessment.

**Design and Setting:**

Data were collected in a cafeteria. Meals, selected by participants (*n*=30), were documented using the AV method: participants video-recorded their tray while audio-recording a description of their selected meal, after which the research team digitally weighed each food item and created an itemized diary record of the food.

**Variables Measured:**

Data from the AV method and from the weighed food diaries were transcribed and entered into a nutrition software analysis program (Nutribase Pro 10.0). Nutrient outputs were compared between the two methods including kilocalories, macronutrients, and selected micronutrients.

**Analyses:**

Using mean scores for each variable, Wilcoxon signed-rank test and Spearman's correlation coefficients were conducted. Interclass correlation coefficient (ICC) was calculated for absolute agreement between the two methods to assess interrater reliability.

**Results:**

With the exception of Vitamin E and total weight, nutrient values were highly correlated between methods and were statistically significant given alpha = 0.05, power = 0.95, and effect size of 0.70.

**Conclusions:**

The AV method may be a meaningful alternative to diary recording in a free-living setting.

## 1. Introduction

Novel methods for assessing nutrient intake in the free-living setting are needed to manage food-related health challenges [1]. The most accurate measure of dietary intake is direct observation and prospective recording of weighed foods [2]. This gold-standard method requires that each item of food be weighed and recorded prior to (pre-meal) and following consumption (post-meal), where the researcher weighs the plate with any leftover food items. This results in the valid and reliable quantification of dietary intake and permits retrospective calculation of nutritional intake (i.e., kilocalories, macro- and micronutrients). However, this method is time-consuming and expensive to execute (e.g., participant/patient training) in research studies and in clinical settings. Furthermore, there is considerable participant burden, and the mere act of keeping such a detailed, weighed food record by participants/parents can become an intervention in and of itself [3].

Historically, the principal methods for assessing dietary intake have included 24-hour recall and food frequency questionnaires (FFQs); but both have been deemed faulty [4]. In fact, Dhurandhar et al. reported that self-reported intakes of energy are regularly used in health research “despite the fact that self-report questionnaires have been repeatedly shown to be seriously flawed [4]” (p. 1110). Three-day food diaries, despite limitations, remain the best option. This method requires participants to record, in detail, all foods and beverages consumed during a three-day time period, ideally, every second day, two during the week and one day during the weekend to capture variability [5]. Limitations include (i) compliance; participants tire of recording food diaries (which is why people are generally recommended not to exceed three days of collection because compliance rates diminish after this timeframe); [6] (ii) self-reporting bias [7]; (iii) poor ability to estimate serving sizes [8]; (iv) the act of writing down one's food fundamentally changes our eating patterns [8]; and (v) the literacy of the person collecting the food data will also fundamentally affect the data provided [8]. The use of food journaling has become mainstream and studied at length in the last decade; however, few advances in this methodology resolving these limitations have occurred. The exception is the use of photography, although this method still requires a written documentation of food items [9].

The advent of digital photography has provided advantages that include its low cost, limited participant burden, and rapid data collection [10]. Additionally, it is possible to extend the method to collect data on populations [11]. Significantly, people have already incorporated this application into their lives as photographing and sharing food photos has become commonplace [12]. Several groups have tested the photographic method and found it to be reliable for food assessment and preferred by participants in research studies [13–18]. Taken together, the literature supports the use of digital photography, provided that the picture resolution is of good quality and under scenarios where the entire meal can be seen [16]. However, in cases where visual estimation was insufficient to determine food choices, food diary data were still required [16].

In 2017, a novel method of assessing food intake in a free-living setting was reported, which removed the requirement of written journaling; specifically, they used audio-video (AV) food recording [19]. This AV method was employed amongst wildland firefighters with the goal to understand food consumption patterns during fire deployment, while eliminating barriers in food data collection. In particular, to achieve compliance amongst participants, it was critical that the food data collection not be overly laborious or time-consuming (i.e., written journal records), or rely on participant memory [20]. Robertson et al. reported that the AV method was beneficial because it could be completed at any time, in any location, and did not impede participant work tasks; written journaling would have been difficult given the nature of their work and the inclement weather and field conditions. The AV method employed by Robertson et al. builds on the principle of the photographic dietary record; that is, the video image provides (presumably) equivalent data to a digital photo but with the benefit that the written journal is no longer required, since participants instead included an audio dictation of the meal and any hidden or unseen ingredients while video-recording [19]. Given that previous research has validated the photographic method for measuring food intake, compared to visual estimation [9, 10], it suggests that video-recording would provide similar results and that the AV method may be a novel, alternative method to estimating food intake via direct observation [21]. However, to our knowledge, the AV method has not yet been validated in the literature. Doing so is meaningful, given the potential applications for this method, specifically, since 91% of the global population has access to portable, personal devices, capable of AV (i.e., cell phones) [22]. Audio-video food journaling could therefore allow people, globally, in a free-living environment, to readily track their food and better understand their food consumption habits. In addition, written journaling also requires a level of literacy; the AV method removes this constraint, increasing participant pools and potentially reaching people, previously unable to contribute food data to the research literature [23]. Given the technological advancement in these devices, including high-resolution video capabilities, we are poised for rapid increases in available, personal food data for analysis, leading to broad-based opportunities for mobile phone interventions designed to support specific components of evidence-based treatments relating to food and health [24, 25].

Therefore, the purpose of this paper was to assess the AV method of recording meals in a free-living scenario, in comparison to direct, weighed food assessment, the gold standard.

## 2. Methods

### 2.1. Participants

Participants were recruited, at random, from a university-based cafeteria. After selecting their meal choices, participants were approached by researchers until a total of *n*=30 participants (female: 18; male: 12) agreed to participate. Forty-seven people were approached, and 17 chose not to participate. Thirty participant meals, including a mix of lunch and breakfast, were documented over a four-hour period, in one day. All meals were included in the data analysis.

Each participant received an incentive of $10.00 for agreeing to participate. Results of this study are solely based on the foods selected, and so no personal data were collected; participant food data were assigned a participant ID number. All participants provided written, informed consent prior to participation, and this study was approved by the Institutional Research Ethics Board (REB#201606100).

### 2.2. Study Design

Data were collected in January 2017, in a cafeteria setting in a medium-sized University in Canada. Each meal was selected by the participant prior to study recruitment, resulting in a range of portion sizes and items chosen per meal.

### 2.3. AV Method

Participants were provided with an iPod touch© (3^rd^ generation) and were asked to AV record the food they selected for their meal; i.e., while video-recording their tray, they provided a verbal description of their food, including a listing of contents (e.g., mustard). The participants were asked if they understood the method and could demonstrate this during a trial recording. Thirty meals were AV recorded in the form of mp4 files. Afterward, in the laboratory, a researcher and a registered dietician, independently, reviewed the AV recordings and created a diary listing of food items for each meal and estimated portion sizes per item indicated in the recording.

### 2.4. Weighted Method

The research assistants digitally weighed participant meals, using sanitary methods. Specifically, the participant was asked to place their food on a clean plate resting on a weigh scale (plate type and weight was premeasured to subtract from the total weight). Each food item was listed and given a weighed value.

## 3. Nutrient Analysis

Participant meals were entered into NutriBase Pro; a software program used by registered dieticians and researchers to analyze the contents of foods and provide an overview of the micro- and macronutrients contained within the meals selected by the participants. Each participant meal was coded and entered into the program three times: (1) AV estimates from researcher; (2) AV estimates from registered dietician; and (3) weighed food items (gold standard).

### 3.1. Audio-Video Method

A researcher explained the AV method using a script and once a participant understood the method, they moved forward in the protocol. Participants selected the video option under the camera setting on the iPod touch©, pressing “start” to begin taking a video of the meal they had selected, while speaking directly into the device, participants dictated the quantities and name of each item on their plate (e.g., one apple) or by volume (e.g., one cup of white rice). In the case of complex items, such as a breakfast sandwich, individual components were listed. Participants would dictate that the sandwich included one fried egg, one white English muffin, one slice of cheddar cheese, and one tablespoon ketchup. The participants were briefly trained on the AV method over a period of approximately 3 to 5 minutes and were asked to demonstrate a “trial” AV recording prior to commencing with a real-time AV recording. The participants were asked if they understood how to perform the AV method correctly, and any clarification required was provided. The AV method requires some level of technological literacy; however, participants in this study did not struggle with this aspect of participation.

### 3.2. Weighted Method

One lead researcher and two research assistants completed all of the data collection and were trained prior to interacting with participants, including sanitary methods of weighing food items. Researchers weighed individual food items on a StarFrit© food scale. Each item was recorded on a coded log sheet, which included a list of items for each selected meal, the weight in grams (g), subject ID, and date and time for the AV recording and weighing. Two research assistants confirmed the weight of each item before it was recorded and then recorded it. The item identified on the coded log sheet, the same as identified by the AV method, was used for data entry.

### 3.3. Nutrient Analysis

All items recorded were entered into the Nutribase Pro nutrition software program. NutriBase draws its nutrient data from the Canadian Nutrient Profile produced by Health Canada [26]. First, the researcher created “client profiles” for each of the video-recordings, using AV subject ID coding to identify each profile. Next, the researcher searched for food items using the “food item search” function and selected the food item from a list of Canadian foods available on Nutribase Pro. At this point, the researcher entered the serving size and repeated this for each food item listed in the AV recording. This develops a nutrient profile with macro- and micronutrient values per food item and for the meal as a whole. This procedure was repeated with estimates provided by (1) the researcher and (2) the registered dietician. There were no discrepancies between item lists.

Next, the researcher followed the same procedure as above, but for the weighed food items. Since the researcher recorded weights during data collection and completed data entry, participant information was coded, and the researcher waited seven days to enter the data in an attempt to prevent recall bias [27]. Again, the researcher developed individual “client profiles” for each of the participants, coded by subject ID. The researcher searched for food items using the “food item search” function and selected the measured serving size. None of the items included in data collection were prepackaged and so the researcher relied solely on developing tailored Personal Food Items (PFI) from the Nutribase Pro 10.0 software.

### 3.4. Statistical Analysis

Prior to data collection, a sample size calculation was performed with the following inputs (type 1 error: 5%; type II error: 90%; effect size: 100 kcal; and 2-sided test). All data are shown as mean ± standard error of the mean (SE). The Shapiro–Wilk test for normality was conducted and outputs indicated that variables were not normally distributed; thus, nonparametric tests were used to compare the weighing methods.

Spearman's correlation coefficient was calculated to determine the strength of the relationship between actual weight of the meals and the averaged AV-estimations from the researcher and the registered dietician. Variable averages were computed for the researcher and registered dietician in order to best capture the weight of each variable given the large amount of variance between each participant. Spearman's correlation coefficient was used to compare the researcher and registered dietician data. Data were expressed as mean ± standard error of the mean (SE), calculated for continuous variables, and compared using Wilcoxon match-pairs signed-rank test. The Wilcoxon match-pairs signed test was used to compare both the weighed and AV recorded methods of dietary measurement. This was followed with the interclass correlation coefficient (ICC) for absolute agreement between the two methods to assess interrater reliability. SPSS statistical package version 20 (SPSS Inc, Chicago, IL) was used for all statistical analysis, reporting significance levels at alpha = 0.05, power = 0.95, and effect size of 0.70.

## 4. Results

Table 1 indicates the mean ± standard deviation for the selected meals estimated by the registered dietician and researcher from the AV recordings; Spearman correlations were calculated. We chose to average these values, since the correlation between them were high. Averaged estimates from the registered dietician and researcher are compared to the actual weight of the meals in Table 2.

The ICC estimates and their 95% confidence intervals were calculated based on a mean-rating (*k* = 3), consistency, and 2-way mixed-effects model. Table 2 represents the weight measured in grams for each variable compared to the average weight, calculated using weighed and registered dietician suggested weight. The results indicate that the researcher and registered dieticians ICC scores were highly correlated for macronutrients and kilocalories and some micronutrients, including sodium, potassium, calcium, zinc, and iron.

From the analysis computed, it was consistently noted that, throughout all analyses, both total weight and vitamin E did not yield significant results. However, all other variables analyzed provided statistically significant results (alpha = 0.05, power = 0.95, and effect size of 0.70).

**Figure 1:**
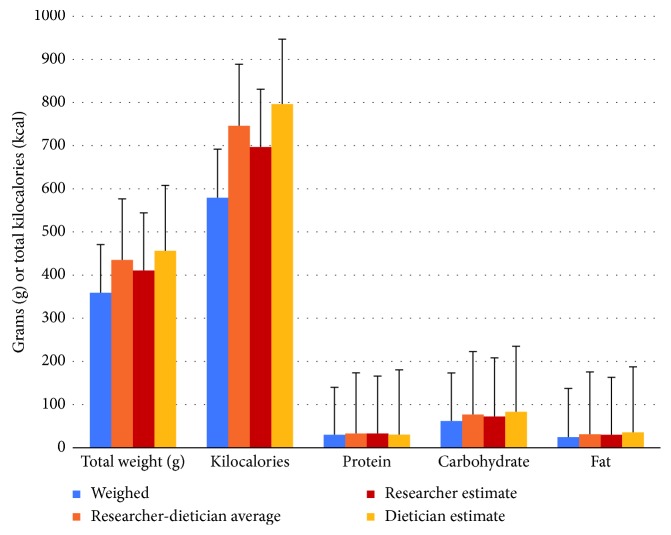
Kilocalories and macronutrients by each method and as estimated by the researcher and the registered dietician.

As seen in Figure 1, the researcher and registered dietician both overestimated portion sizes, resulting in higher macronutrient and kilocalorie outputs per meal, compared to the macronutrient and kilocalorie outputs from the actual weight of the food items.

## 5. Discussion

This validation study was aimed at assessing a novel method of calculating dietary intake in a free-living setting, which is more practical than previous methods. The results from this study suggest that the audio-video method is a valid method for providing visual and audio information of food selection, allowing a researcher or RD to make accurate estimations of food to determine energy consumption; replacing the laborious nature of food journaling, since it is correlated to precise weights of food items.

### 5.1. Total Weight

The AV videos were assessed after data were collected to provide serving size estimates of the items recorded for each meal. After the data were entered to Nutribase Pro from both the researcher and the registered dietician, outputs were averaged and then descriptive statistics were run on those averages values. This is likely the cause of the discrepancy between actual weight and the video estimations by the researcher and registered dietician.

### 5.2. Kilocalories and Nutrients

Unlike other cafeteria-based studies [20], participants in the present study served their own meals, none of which were preportioned by trained cafeteria staff. Likewise, a list of ingredients and cooking methods was not provided by the cafeteria management company, and each meal was presented to the researchers after selection by the participant. Given that the normal application of the gold-standard method would not have access to these conditions, we wanted to compare the nutrient values produced from assessing the AV method to the use of the weighed method. While the spearman correlation demonstrates a moderate to very good level (*r* values ranging from 0.50 to 0.75 indicate moderate to good correlation, and *r* values from 0.75 to 1 point to very good to excellent correlation between the variables) of correlation between the two diary methods, suggesting that the nutrient values produced from assessing the AV method are valid, and the mean standard deviation reflects that participants selected varying levels of total calories per meal. This is critical because it demonstrates that the AV method is able to capture differences in portion size, resulting in a range of caloric values per meal, even in a free-living setting.

The ability to accurately estimate portion sizes is important to understanding actual calories consumed, reducing the margin of error in a food journal. The researcher and registered dietician estimated portion sizes in this study because it is known that trained persons can more accurately estimate portion sizes when compared to nontrained persons [28], and in the present study, participants inaccurately estimated portion sizes or commented in their recordings that they did not know how to assess portion sizes, saying “I'm not exactly sure, maybe 1 cup, could be more though”. Some researchers have recommended portion size training methods for participants to help them accurately estimate portion sizes, resulting in improved portion size estimation accuracy [28, 29]. Since weight gain is directly attributed to an overconsumption of calories compared to energy expenditure, it is critical to improve current methods of portion size estimation by the general public, in a free-living setting. One solution to this problem is the introduction of computer-based estimation using image recognition software. In this study, both the researcher and registered dietician overestimated portion sizes, reinforcing the need for unbiased, computer-based estimation in the free-living setting, where weighing individual food items is not an option.

Some aspects of the present study could be considered limitations and merit future methodological modifications. First, since participants tend to inaccurately estimate portion sizes, we did not rely on, nor ask for participant estimates. Since one aim of the study was to validate the AV method, the researcher and registered dietician estimated portion sizes because they are known to be more accurate estimations [28]. However, there is a cost associated with requiring a registered dietician or trained individual to evaluate food items from the AV recordings and use a nutrient database. Nutrient database subscriptions come at a cost and are not likely to be used and purchased by untrained individuals. One alternative is for individuals to use free, online nutrient data-sharing programs, such as “My FitnessPal.” In a clinical setting, registered dieticians perform analyses and have fee-for-service appointments to provide nutrition counseling. The cost of these services is typically paid for by the patient or client or through their health coverage. Compared to written food journals, it is likely that the same or similar costs would be incurred given that a registered dietician or trained individual would still need to enter the food values in a nutrient database to determine nutrient outputs. Second, the food captured in the present study was done in a cafeteria setting, which is different from the home or other free-living environments. In a cafeteria, participants can only select foods that are made available to them, and signage related to, and presentation of food items may influence a participants' food selection, which may not reflect “typical” foods in their diet.

The AV method was designed based on previous success with photographic food journals. When compared to traditional methods of three-day food records, the use of digital photography for assessing food choices has been shown to be preferred amongst participants [17] and as accurate as real-time estimates of food [18]. There is however an inherent challenge to using this methodology: it is difficult/impossible to determine food contents when the food is not readily visible. Gauthier et al. [9] overcame this problem this by having participants list the items in their meals; however, this created similar challenges found with written journaling. The use of audio-video recording of meals therefore aimed to reduce the burden of participation and facilitated food selection such that participants may also have decreased awareness about the food assessment process. Participants “chatted” into the iPod, highlighting those components of the meals, which they perceived would be difficult to view. They were not required to estimate portion size nor were they required to itemize the foods in their meal. Therefore, for some meals, participants merely verbalized their beverage. We identify several additional advantages to this method including (i) using the same tools already employed for digital photography; (ii) excellent resolution on iPods, iPhones, and other devices that already exist; (iii) video adds dimension to the photos (i.e., several angles can be captured simultaneously, while freeze-framing for portion analysis); and (iv) audio-video allows the participant to speak directly to the researcher describing the meal and its components, rather than diary keeping and audio can be transcribed verbatim. In addition, since participants are not being asked to estimate the serving sizes or number, they may be more accurately depicting real choices, rather than modifying behaviour; this requires further research to determine. Lastly, people are already photographing their food and electronically sending food pictures, suggesting that they are comfortable with this type of data collection methodology. As some may find dictating information uncomfortable in a public setting, developing image recognition software should be explored, as it would eliminate the need to dictate food items and unseen items.

## 6. Implications for Future Research and Practice

In the clinical setting, registered dieticians frequently ask patients to keep a food diary to assess their food behaviours, to help advise them about food choices [30], or as a method of self-regulation or self-monitoring in an effort to improve eating patterns. Understanding energy expenditure and energy intake is complicated for the average person, and so one goal of utilizing a food journal is to keep track of approximate portions of food and more specifically calories, fat, protein, and carbohydrate; however, multiple challenges are experienced with this approach. First, patients tire of recording food diaries [6]; the AV method reduces participant burden and therefore may improve extended food collections. Second, patients tend to be biased when self-reporting [7]; the AV method reduces the capacity to do this, as it is literally “showing” the foods selected. Third, patients are poor at estimating their serving sizes [8]; although this study demonstrates that even the experts are not perfect at estimating size given the discrepancy in estimations made, it is more likely that the registered dietician or researcher would generally estimate serving sizes better than the untrained client. Fourth, given that food journaling is one method for weight loss, we know that writing down one's food fundamentally changes our eating patterns [8]; we do not know whether this is true for AV journaling. Lastly, the AV method bypasses literacy problems in food data collection [8]. As indicated, the AV method requires some level of technological literacy. However, following a brief tutorial on the AV method, all participants were able to perform the AV method as required. Overall, this method provides significant advantages over written food journals and should be considered for future research and clinical applications.

Accurate diet analysis of macro- and micronutrient intake allows healthcare providers, including registered dieticians, to provide accurate counseling to improve and maintain health. In fact, AV journaling may provide the clinician additional information which could be used to help counsel the patient (e.g., time-of day when the meal is consumed).

## 7. Conclusion

Diet intervention is a critical community issue for many Canadians; a new method of food journaling, which includes an easy to use application for the general public to use in a free-living environment and enhances communication between clients and healthcare providers, is needed. The AV method allows participants with limited health literacy and language and literacy barriers to participate in meal recording for diet analysis for the purpose of dietetic consultation and is clinically comparable to the gold-standard weighed method. How the AV method compares to written journaling is not yet known and should be explored in future research, given that this is the most common type of diet recording used in practice [31].

## Figures and Tables

**Table 1:** Software analysis data for estimates by researcher and registered dietician for macro- and micronutrients.

	Researcher (mean ± SD)	Registered dietician (mean ± SD)	Spearman correlation
Total weight (g)	411.6 ± 139.05	456.5 ± 195.68	0.599
Calories (kcal)	697.5 ± 352.64	794.5 ± 388.88	0.774
Energy (kJ)	2823.4 ± 1314.16	3255.0 ± 1689.37	0.781
Protein (g)	31.5 ± 15.30	30.8 ± 14.79	0.667
Carbohydrates (g)	74.4 ± 44.09	85.5 ± 54.33	0.803
Fiber (g)	5.8 ± 4.75	7.0 ± 5.78	0.798
Fat (g)	29.8 ± 19.52	35.1 ± 19.25	0.675
Saturated fat (g)	7.7 ± 5.01	8.1 ± 3.67	0.731
Trans fat (g)	2.9 ± 9.46	0.7 ± 749	0.513
Vitamin A (*µ*g)	230.7 ± 220.19	239.9 ± 204.65	0.551
Vitamin C (mg)	20.7 ± 19.20	23.9 ± 27.21	0.453
Vitamin D (mg)	52.3 ± 72.59	57.4 ± 69.62	0.533
Vitamin E (mg)	5.2 ± 3.84	5.1 ± 3.52	0.596
Calcium (mg)	215.7 ± 228.16	215.7 ± 174.19	0.778
Magnesium (mg)	83.0 ± 51.71	83.2 ± 48.57	0.670
Potassium (mg)	916.4 ± 551.26	978.3 ± 700.62	0.769
Sodium (mg)	1355.8 ± 893.43	1203.2 ± 695.19	0.817
Iron (mg)	4.3 ± 1.82	4.7 ± 2.15	0.745
Zinc (mg)	3.4 ± 2.10	3.6 ± 1.95	0.818
Folate (*µ*g)	114.0 ± 71.15	124.7 ± 81.12	0.671

**Table 2:** Macro- and micronutrient comparisons between methods.

	Weighed mean ± SD	AV method (R/RD) (mean ± SD)	ICC	Wilcoxon signed-ranks
Total weight (g)	359.3 ± 121.79	434.1 ± 167.36	0.793	*Z*=−3.013, *p*=0.003
Calories (kcal)	593.2 ± 265.74	746.0 ± 370.76	0.813	*Z*=−3.198, *p*=0.001
Energy (kJ)	2477.8 ± 1110.64	3039.2 ± 1501.76	0.808	*Z*=−2.900, *p*=0.004
Protein (g)	29.4 ± 14.49	31.2 ± 15.04	0.891	*Z*=−1.386, *p*=0.166
Carbohydrates (g)	63.2 ± 30.07	79.9 ± 49.21	0.793	*Z*=−2.865, *p*=0.004
Fiber (g)	5.0 ± 2.89	6.4 ± 5.27	0.851	*Z*=−2.779, *p*=0.005
Fat (g)	25.4 ± 14.81	32.5 ± 19.39	0.808	*Z*=−2.922, *p*=0.003
Saturated fat (g)	6.4 ± 4.23	7.9 ± 4.34	0.900	*Z*=−2.916, *p*=0.004
Trans fat (g)	1.6 ± 6.25	1.8 ± 5.10	0.804	*Z*=−0.743, *p*=0.457
Vitamin A (*µ*g)	229.1 ± 216.03	235.3 ± 212.42	0.875	*Z*=−0.761, *p*=0.447
Vitamin C (mg)	17.7 ± 18.04	22.3 ± 23.21	0.823	*Z*=−1.395, *p*=0.163
Vitamin D (mg)	48.7 ± 66.92	54.9 ± 71.11	0.859	*Z*=−2.847, *p*=0.004
Vitamin E (mg)	3.1 ± 2.76	5.1 ± 3.68	0.586	*Z*=−2.392, *p*=0.017
Calcium (mg)	202.1 ± 223.47	215.7 ± 201.18	0.911	*Z*=−2.222, *p*=0.026
Magnesium (mg)	68.9 ± 24.05	83.1 ± 50.14	0.778	*Z*=−1.564, *p*=0.118
Potassium (mg)	785.2 ± 350.49	947.3 ± 625.94	0.821	*Z*=−1.224, *p*=0.221
Sodium (mg)	1114.8 ± 627.64	1279.5 ± 794.31	0.880	*Z*=−1.142, *p*=0.254
Iron (mg)	3.8 ± 1.85	4.5 ± 1.99	0.885	*Z*=−2.596, *p*=0.009
Zinc (mg)	3.3 ± 2.08	3.5 ± 2.03	0.976	*Z*=−2.147, *p*=0.032
Folate (*µ*g)	105.9 ± 68.35	119.4 ± 76.16	0.933	*Z*=−2.006, *p*=0.045

## Data Availability

The data used to support the findings of this study are available from the corresponding author upon request.
